# The association of serum uric acid with cognitive impairment and ATN biomarkers

**DOI:** 10.3389/fnagi.2022.943380

**Published:** 2022-07-18

**Authors:** Shan Huang, Jun Wang, Dong-Yu Fan, Tong Luo, Yanli Li, Yun-Feng Tu, Ying-Ying Shen, Gui-Hua Zeng, Dong-Wan Chen, Ye-Ran Wang, Li-Yong Chen, Yan-Jiang Wang, Junhong Guo

**Affiliations:** ^1^Department of Neurology, First Affiliated Hospital, Shanxi Medical University, Taiyuan, China; ^2^First Clinical Medical College, Shanxi Medical University, Taiyuan, China; ^3^Department of Neurology, Daping Hospital, Third Military Medical University, Chongqing, China; ^4^Chongqing Key Laboratory of Ageing and Brain Diseases, Chongqing, China; ^5^Shigatse Branch, Xinqiao Hospital, Third Military Medical University, Shigatse, China; ^6^Department of Biomedical Engineering, Chongqing University, Chongqing, China; ^7^Department of Anaesthesiology, Daping Hospital, Third Military Medical University, Chongqing, China; ^8^State Key Laboratory of Trauma, Burn and Combined Injury, Institute of Surgery Research, Daping Hospital, Third Military Medical University, Chongqing, China; ^9^Center for Excellence in Brain Science and Intelligence Technology, Chinese Academy of Sciences (CAS), Shanghai, China

**Keywords:** Alzheimer’s disease, uric acid, Aβ42, tau, cognitive impairment

## Abstract

**Background:**

Cognitive impairment (CI) has become a worldwide health problem. The relationship between CI and uric acid (UA) is contradictory.

**Objective:**

We included participants with a full spectrum of CI, from cognitively unimpaired (CU) to dementia, from the Chongqing Ageing & Dementia Study (CADS).

**Methods:**

First, we identified the relationships between serum UA (sUA) and cognitive function in different stages of CI. Second, we analyzed these relationships among different stages and types of CI. Finally, we explored the association between sUA and amyloid/tangle/neurodegeneration (ATN) biomarkers.

**Results:**

We recruited 427 participants from the CADS, including 382 participants with mini-mental state examination (MMSE) evaluation. The levels of sUA were positively correlated with MMSE scores (*p* < 0.001), and the correlation was prominent in the course of dementia and in the type of Alzheimer’s disease (AD). The levels of UA had a positive correlation with plasma amyloid-β 42 (Aβ42) (*p* = 0.004). Higher levels of sUA weakened the correlation of MMSE scores with CSF ATN biomarkers and the correlation of CSF Aβ42 with tau.

**Conclusion:**

UA is positively correlated with cognitive function, especially in the advanced stage of AD. The probable neuroprotective effects of sUA mainly act on Aβ42 and the downstream pathological cascade.

## Introduction

Dementia is currently the seventh leading cause of death among all diseases and one of the major causes of disability and dependency among older people. It is estimated that more than 55 million people live with dementia, and the number could rise to 78 million by 2030 and to 139 million by 2050 ([WHO], 2021b). Alzheimer’s disease (AD) is the most common type of age-related dementia, accounting for 60-70% of cases ([WHO], 2021a). However, the pathogenesis is not fully understood, and there is a lack of early diagnosis and effective therapy (Jia et al., 2020).

Oxidative damage is increasingly aggravated during aging and is an important pathological process of cognitive impairment (CI) and AD; in turn, oxidative damage can accelerate aging and AD development. The imbalance between the production of oxidative stress reactive oxygen species (ROS) and reactive nitrogen species (RNS), and antioxidant can contribute to the pathology of AD (Butterfield and Halliwell, 2019). For examples, oxidative damage could lead to the accumulation of amyloid-abeta (Aβ), neurofibrillary tangles, synaptic damage and microglia response (Cheignon et al., 2018; Buccellato et al., 2021).

Uric acid (UA), the final metabolite of purine, is regulated by diet intake, metabolism and renal excretion. UA is a natural antioxidant in the human body that scavenges oxygen free radicals. For examples, it can improve the removal of ROS, RNS, peroxyl radicals and hydrogen radicals and reduce the pathological process associated with oxidative stress in circulation, such as autoxidation of hemoglobin, lipid peroxidation and peroxide production by macrophage (Becker et al., 1991; Sautin and Johnson, 2008).

It has been reported that UA can prevent aging and age-related diseases (Becker et al., 1991; Sautin and Johnson, 2008). Thus, the relationship between UA and dementia has gained increasing attention. Some studies have suggested that serum UA (sUA) is a protective factor of cognition and is decreased in AD patients compared with cognitively unimpaired (CU) individuals (Polidori and Mecocci, 2002; Kim et al., 2006; Cankurtaran et al., 2013; Al-khateeb et al., 2015; Tana et al., 2018; Boccardi et al., 2021). Prospective studies have suggested that higher baseline sUA levels are associated with a lower risk of developing dementia and mild cognitive impairment (MCI) (Wang et al., 2017; Scheepers et al., 2019; Chen et al., 2021). However, UA could also be prooxidative and proinflammatory under certain conditions (So and Thorens, 2010; Kimura et al., 2021). Other studies have suggested that sUA may aggravate amyloidosis burden in the brain and have found higher sUA levels in participants with preclinical AD or mild-moderate AD (Zuliani et al., 2018; Li et al., 2021). We investigated the underlying reasons for the contradictory results and found that participants in these studies were in different disease stages, including preclinical, MCI, and dementia. In addition, the subtypes of dementia were different in these studies.

To investigate whether sUA functions and changes differently in different stages and types of CI, we observed the dynamic changes in sUA levels during the whole progression of AD and the relationship of sUA with different types of dementia. Furthermore, we analyzed the relationship of sUA and amyloid/tangle/neurodegeneration (ATN) biomarkers in a Chinese cohort.

## Materials and methods

### The inclusion and exclusion criteria of participants

This study was based on data from the Chongqing Ageing & Dementia Study (CADS). CADS is an on- going cohort study based on the southwest Chinese population and from Daping Hospital, aimed to explore the evolution mechanisms of ageing to AD and identify biomarkers of diagnosis and intervention for AD. Participants were recruited from CADS between January 2012 and January 2021. Participants were included with clinical evaluations, neuropsychological evaluations (mini-mental state examination (MMSE) evaluations, clinical dementia rating (CDR) scores etc.), and serum renal function biomarkers (including sUA) data. Participants were excluded for the following reasons: concomitant neurological disorders, including symptomatic stroke, intracranial infection, multiple sclerosis, Parkinson’s disease, epilepsy, metabolic encephalopathy, hydrocephalus etc.; psychiatric disorders, including schizophrenia, depression etc.; severe systematic disorders, including tumors, severe liver insufficiency, severe renal insufficiency and severe infection; refusal of blood sampling or lumbar puncture. This study was approved by the Institutional Review Board of Daping Hospital, and all participants and their caregivers provided informed consent.

### Cerebrospinal fluid sampling and testing

Participants received lumbar puncture after a fast following the standard procedure, and cerebrospinal fluid (CSF) samples were collected in polypropylene tubes and centrifuged at 2,000 *g* and 4°C for 10 min (Blennow et al., 2010). These processed CSF samples were stored frozen at −80°C until used. The levels of CSF amyloid-β 40 (Aβ40), amyloid-β 42 (Aβ42), total tau (T-tau) and phosphorylated tau-181 (P-tau181) were analyzed using commercially available enzyme-linked immunosorbent assay (ELISA) kits (INNOTEST, Fujirebio Europe, Ghent, Belgium) (Carrillo et al., 2013). CSF neurofilament light chain (NFL) was analyzed using a single molecule array (SIMOA) onboard an automated SIMOA HD-1 analyzer (Quanterix United States). Preanalytical process and quality control strictly followed protocols from the Alzheimer’s Association guidelines, and our laboratory is one participant of the quality control (QC) program (code Lab129)(Mattsson et al., 2011, 2013; Hansson et al., 2021). Informed consent was obtained before sampling CSF, and the interval among CSF sampling and clinical evaluation was shorter than two weeks.

### Blood sampling and testing

Blood samples were collected for the analysis of AD core biomarkers and basic tests, including renal function biomarkers. Fasting blood was collected between 07:00 a.m. and 09:00 a.m. A portion of blood samples was centrifuged within an hour of collection, and ethylenediaminetetraacetic acid plasma was aliquoted in 0.5-ml polypropylene tubes and stored at −80°C until the measurements of plasma AD core biomarkers (Aβ40, Aβ42, and T-tau) by a SIMOA Human Neurology 3-PlexA assay kit (Quanterix, United States) onboard the automated SIMOA HD-1 analyzer (Quanterix United States). Another portion of blood samples was used to measure basic tests, including apolipoprotein E (*APOE*) genotypes and sUA. *APOE* genotypes (rs429358 and rs7412) were determined by the polymerase chain reaction restriction fragment length polymorphism method. The sUA was measured on a Beckman AU-5800 instrument (Beckman Coulter, Inc., United States) by the uricase-peroxidase method (Reportable range: 89-1785 μmol/L; Sensitivity: the absorbance change value range was between 0.05 and 0.13 with a concentration of 314 μmol/L; Repeatability: the coefficient of variation of quality control was <5.0%; Inter-batch variability: the relative range of quality control between batches was <10.0%), and the biological reference intervals were 208.0-428.0 μmol/L for males and 155.0-357.0 μmol/L for females. Informed consent was obtained before sampling blood, and the interval among blood sampling and clinical evaluation was shorter than two weeks.

### Cognitive assessment and Alzheimer’s disease diagnosis

Cognitive function was evaluated by adjusted MMSE scores and CDR scores. CU is defined as scores ≥28 for middle school education and above, scores ≥24 for primary school education and scores ≥21 for illiteracy (Folstein et al., 1975); MCI is defined as scores 25-27 for middle school education and above, scores 21-23 for primary school education and scores 18-20 for illiteracy, and MCI corresponds to the prodromal stage of dementia (Petersen et al., 1999; Winblad et al., 2004); dementia is defined as scores ≤24 for middle school education and above, scores ≤20 for primary school education and scores ≤17 for illiteracy (Folstein et al., 1975). CDR scores are used to evaluate the severity of dementia, and mild dementia is defined as scores = 1; moderate dementia is defined as scores = 2; and severe dementia is defined as scores = 3 (Morris, 1993).

Clinical diagnosis of AD was made according to the recommendations from the National Institute on Aging-Alzheimer’s Association workgroups on diagnostic guidelines for Alzheimer’s disease in 2011 (McKhann et al., 2011; Jack et al., 2012). In CADS, CSF Aβ42 ≤ 933 pg/mL (A +), CSF phosphorylated tau181 (P-tau181) > 48.7 pg/mL (T +), and CSF total tau (T-tau) >313 pg/mL (N +) were used to define abnormal amyloid, tangle levels or neurodegeneration in the brain.

### Statistical analysis

Normally distributed continuous variables are presented as the mean (standard deviation, SD), and non-normally distributed continuous variables are presented as the median (interquartile range, IQR). Categorical variables are presented as frequencies (percentages). Student’s *t*-test and analysis of variance were used to compare normally distributed continuous data across groups, and the Mann–Whitney *U* test and Kruskal–Wallis test were used to compare non-normally distributed continuous data. Regression analysis was used to identify the independent influencing factors. Analysis of covariates or multivariable model by regression analysis was used for adjusting the covariates of sUA after *t*-test or Mann–Whitney *U* test. The chi-squared test was used for categorical data. Pearson’s (normal distribution) or Spearman’s (non-normal distribution) analysis was used to evaluate correlations, and partial correlation analysis was used to adjust the covariates of sUA. Hierarchical regression analysis were used to evaluate the moderating effect. All hypothesis testing was two-sided, and *p*<0.05 was considered statistically significant. All statistical analyses were performed using R programming language (version 4.1.1) and SPSS software (version 26.0, Inc., Chicago, United States).

## Results

### Demographic characteristics and uric acid-related covariables

**FIGURE 1:**
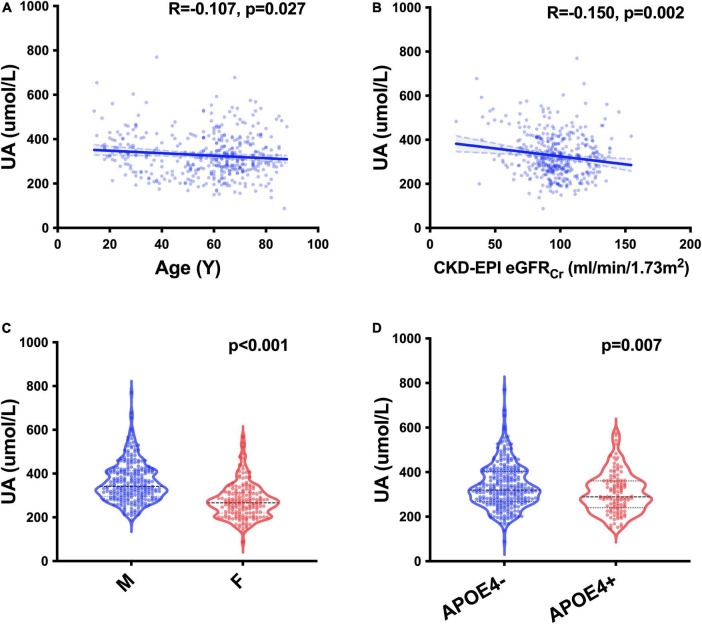
UA-related factors. Spearman’s correlation analysis between the levels of sUA and age **(A)** and CKD-EPI eGFR_Cr_ **(B)**; Mann–Whitney U test of the levels of sUA grouped by sex **(C)** and the status of *APOE* ε4 carriers **(D)**.

We recruited 427 participants from the CADS, including 382 participants with MMSE evaluation (Table 1). We screened UA-related covariables and found that age (*p* = 0.027), sex (*p* < 0.001), *APOE* gene (*p* = 0.007) and glomerular filtration rate (GFR) estimated by the Chronic Kidney Disease Epidemiology Collaboration creatinine equation (CKD-EPI eGFR_Cr_) (p = 0.002) were related to sUA (Figure 1). Regression analysis suggested that the four covariables were independent influencing factors of sUA. In our cohorts, sUA had no correlation with hypertension, diabetes, dyslipidaemia or coronary heart disease (CHD).

**TABLE 1:** Characteristics of the cohorts from CADS.

Characteristics	CU (*n* = 186)	MCI (*n* = 71)	Dementia (*n* = 125)	*p*
**Demographic characteristics and clinical results**				
Age, median (IQR), years	41(28-62)	66 (62-73)	68 (61-75.5)	< 0.001
Male, *n* (%)	128(68.8)	54(76.1)	63(50.4)	< 0.001
Weight, median (IQR), Kg	61(55-70)	61(55-69)	60(51.9-66)	0.039
*APOE* ε4 carriers, *n* (%)	37(21.1)	18(26.9)	44(36.7)	0.013
Education, median (IQR), years	12(12-15)	9(9-12)	9(6-12)	< 0.001
MMSE scores, median (IQR)	29(28-30)	26(25-27)	16(12-21)	< 0.001
GFR, median (IQR), ml/min/1.73 m^2^	109(95-123)	91(84-99)	91(81-99)	< 0.001
UA, median (IQR), μmol/L	330(275-399)	315(269-393)	292(247-347)	0.005
**Medical history**				
Diabetes, *n* (%)	12(6.6)	8(11.9)	19(18.3)	0.010
Hypertension, *n* (%)	29(16)	21(30.9)	36(35.6)	< 0.001
Dyslipidemia, *n* (%)	2(1.1)	4(6)	6(6)	0.045
CHD, *n* (%)	8(4.4)	9(13.4)	18(17.5)	0.001
**CSF AD core biomarkers**				
Aβ40, median (IQR), pg/mL	10175(7476-14153)	12355(9223-14461)	11241(7476-15195)	0.107
Aβ42, median (IQR), pg/mL	1396(1169-1650)	1433(1052-1646)	803(558-1386)	< 0.001
T-tau, median (IQR), pg/mL	124(92-174)	165(122-242)	225(142-422)	< 0.001
P-tau, median (IQR), pg/mL	35.4(27.5-46.1)	45.2(39.0-55.2)	57.0(42.3-74.2)	< 0.001
NFL, median (IQR), pg/mL	737(495-1153)	1500(1066-1990)	1360(1068-2419)	< 0.001
**Plasma AD core biomarkers**				
Aβ40, median (IQR), pg/mL	222(138-260)	237(174-307)	218(165-276)	0.305
Aβ42, median (IQR), pg/mL	11.4(8.58-15.4)	13.2(10.3-16.1)	11.5(8.6-13.5)	0.065
T-tau, median (IQR), pg/mL	2.13(1.57-4.10)	3.83(2.68-5.49)	3.07(2.14-4.87)	0.046

IQR, interquartile range; APOE ε4, apolipoprotein E ε4 allele; MMSE, mini-mental state examination; GFR, Chronic Kidney Disease Epidemiology Collaboration creatinine equation in estimating glomerular filtration rate; UA, uric acid; CHD, coronary heart disease; CSF, cerebrospinal fluid; AD, Alzheimer’s disease; Aβ, amyloid-beta; T-tau, total tau; P-tau181, phosphorylated tau181; NFL, neurofilament light chain.

### The relationships of uric acid and cognitive function

**FIGURE 2:**
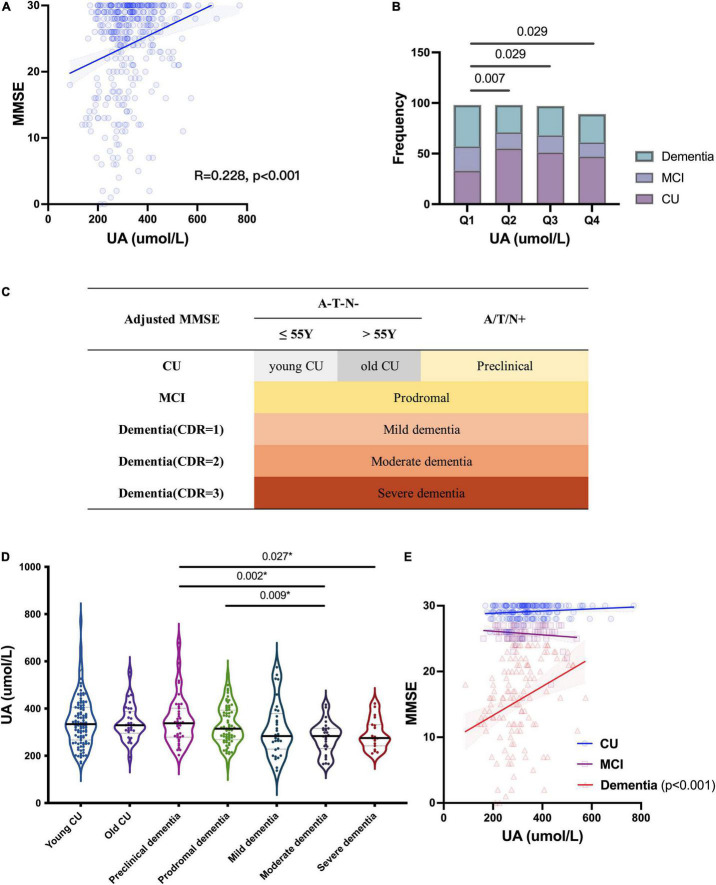
Relationship of UA and cognitive function. **(A)** Spearman’s correlation analysis between the levels of sUA and MMSE scores. **(B)** Proportion of CI phrases in different levels of sUA (Q1 - Q4: the levels of sUA were stratified into 4 subgroups from the lowest to the highest levels as quartiles 1–4). **(C)** Staging as MMSE, CDR and cut-offs of ATN biomarkers; A/T/N + means positive on at least one ATN biomarkers. **(D)** Kruskal–Wallis test for the levels of sUA among the different cognitive stages. The p value, two-tailed Mann–Whitney *U* test between two subgroups; **(E)** Spearman’s correlation analysis between the levels of sUA and MMSE scores in the stages CU, MCI and dementia. The p value, two-tailed chi-squared test.

To investigate the relationship between UA and cognition, we first analyzed the correlation between the levels of sUA and MMSE scores, and there was a positive correlation between them (R = 0.228, *p* < 0.001) (Figure 2A). The correlation remained statistically significant after adjustment (*p* = 0.039). The levels of sUA were stratified into 4 subgroups from the lowest to the highest levels, and subgroups 1-4 corresponded to quartiles 1–4 (Q1 - Q4) of the sUA levels (male: (0, 296), [296, 341.9), [314.9, 412.7), [412.7, ∞) μmol/L; female: (0, 219.4), [219.4, 266.7), [266.7, 315.2), [315.2, ∞) μmol/L). We compared the proportion of CI phases among the subgroups of sUA from Q1 to Q4, and the proportion of dementia and MCI in Q1 (the lowest levels of sUA) was significantly higher than that in Q2 (*p* = 0.007), Q3 (*p* = 0.029) and Q4 (*p* = 0.029) (Figure 2B).

### Uric acid in different stages of cognitive impairment

Then, we presented sUA in the different stages of CI (Figure 2C). There is a dynamic decline in sUA levels with the progression of CI (Figure 2D). The levels of sUA were lower in moderate dementia than in preclinical dementia (*p* = 0.002) and MCI (*p* = 0.009) and lower in severe dementia than in preclinical dementia (*p* = 0.027) (Figure 2D). These differences were still statistically significant after adjustment (*p* = 0.003, 0.026, 0.029, respectively). We further verified the correlation between UA and cognition in different cognitive stages, and the positive correlation was more significant in dementia (R = 0.382, *p* < 0.001) than in the two earlier stages (Figure 2E).

### Uric acid in different types of cognitive impairment

**FIGURE 3:**
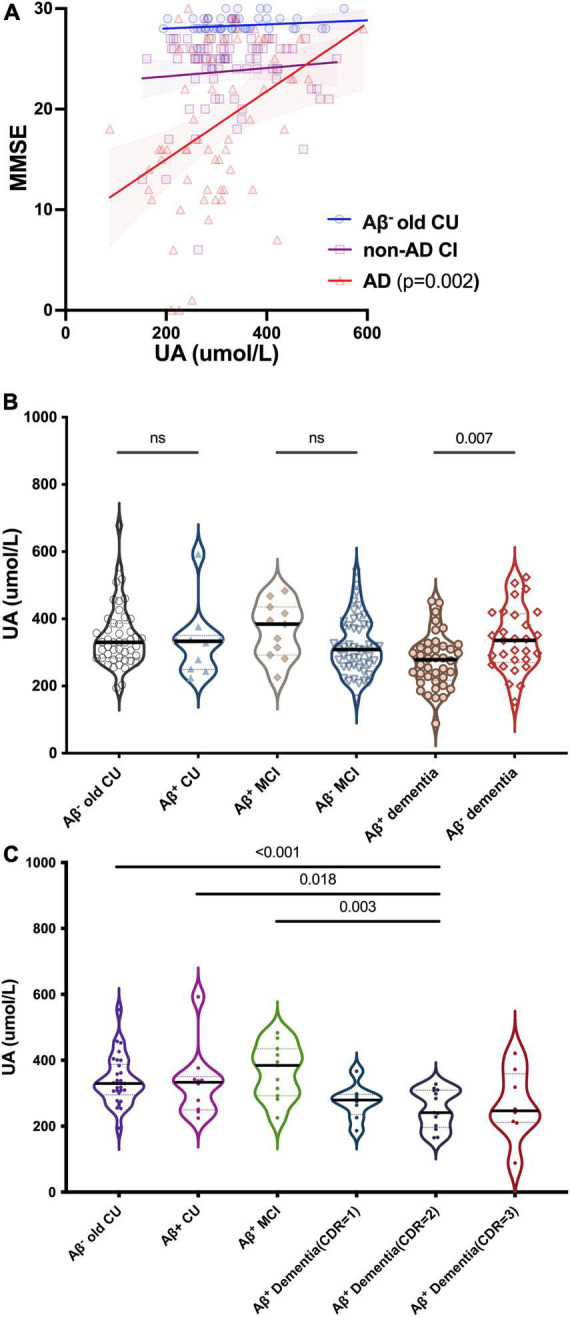
Relationships between UA and cognition in CU and different types of CI. **(A)** Spearman’s correlation analysis between the levels of sUA and MMSE scores in old CU, non-AD CI and AD. **(B)** Kruskal–Wallis test for the levels of sUA between cognitive subgroups. **(C)** Dynamic changes of sUA along for the process of AD. The *p* value, two-tailed Spearman’s correlation **(A)** or Mann–Whitney *U* test **(B,C)**. ns, not significant.

We divided participants into different types of CI: CU contained Aβ^–^ young CU and Aβ^–^ old CU; AD contained Aβ^+^ CU (preclinical AD), Aβ^+^ MCI (prodromal AD) and Aβ^+^ dementia; Non-AD CI contained Aβ^–^ MCI and Aβ^–^ dementia. MMSE scores were positively correlated with sUA in AD (*R* = 0.398, *p* = 0.002) but not in Aβ^–^ old CU (*R* = 0.292, *p* = 0.118) or non-AD CI (*R* = 0.095, *p* = 0.334), and the positive correlation was still significant after adjustment (*p* = 0.013) (Figure 3A). The levels of sUA were lower in the Aβ^+^ dementia subgroup than in the Aβ^–^ dementia subgroup (*p* = 0.007) (Figure 3B). Throughout the process of AD, we found that sUA levels first increased and then decreased, and the peak was in the stage of MCI (Figure 3C). The levels of sUA were lower in Aβ^+^ moderate dementia than in old CU (*p* < 0.001), Aβ^+^ CU (*p* = 0.018) and Aβ^+^ MCI (*p* = 0.003). These differences were still statistically significant after adjustment (*p* = 0.014, 0.018, 0.023, respectively).

### Association between uric acid and ATN biomarkers

**FIGURE 4:**
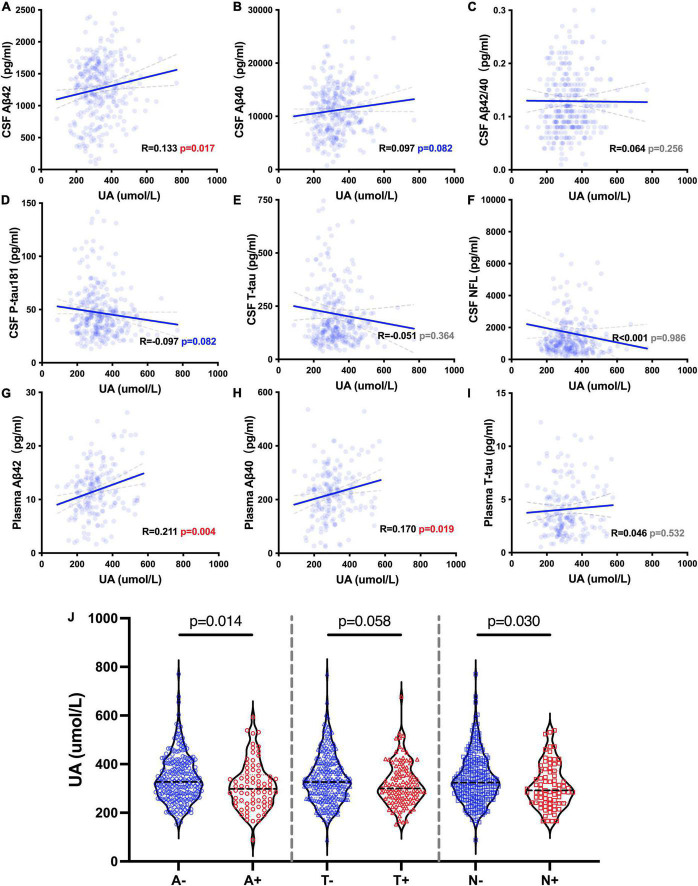
UA-related ATN biomarkers. **(A–I)** Spearman’s correlation analysis between the levels of sUA and ATN biomarkers. **(J)** Mann–Whitney *U* test between groups according to the cut-offs of CSF ATN biomarkers, including A– and A +, T– and T + and N– and N +.

We analyzed the correlations between the levels of sUA and AD biomarkers in CSF and plasma. The levels of sUA had positive correlations with the levels of CSF Aβ42 (*R* = 0.133, *p* = 0.017), plasma Aβ42 (*R* = 0.211, *p* = 0.004) and plasma Aβ40 (*R* = 0.170, *p* = 0.019) (Figures 4A,G,H). There were no significant correlations between the levels of sUA and CSF Aβ40 (*p* = 0.082), CSF Aβ42/Aβ40 (*p* = 0.256), CSF P-tau181 (*p* = 0.082), CSF T-tau (*p* = 0.364), CSF NFL (*p* = 0.986) and plasma T-tau (*p* = 0.532) (Figures 4B-F,I). The difference in the correlation between plasma Aβ42 and sUA was still statistically significant after adjustment (*p* = 0.019). We also compared the levels of sUA grouping by the cut-offs of the ATN biomarkers. The levels of sUA were lower in A + than A− (*p* = 0.014) and in N + than N− (*p* = 0.030), and there were no significant differences in T + and T− (*p* = 0.058) (Figure 4J).

**FIGURE 5:**
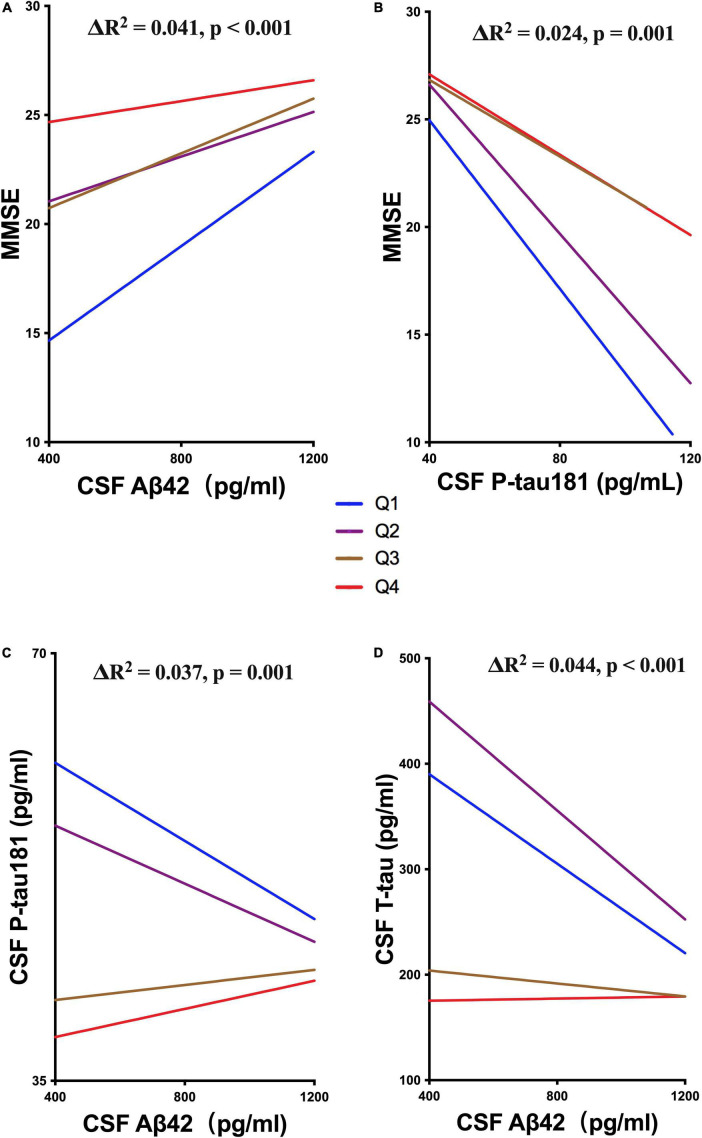
Moderating effect of UA and ATN biomarkers. **(A,B)** Hierarchical regression analysis of the moderating effect of the sUA levels in the association of CSF Aβ42 and P-tau181 with MMSE scores. **(C,D)** Hierarchical regression analysis of the moderating effect of the sUA levels in the association of CSF Aβ42 with P-tau181 and T-tau.

We also explored whether UA moderated cognitive function by interacting with ATN biomarkers. The levels of sUA were stratified into 4 subgroups from the lowest to the highest levels as described above (Q1 – Q4). The analyses showed that high levels of sUA alleviated the association of CSF Aβ42 with MMSE scores (ΔR^2^ = 0.041, *p* < 0.001) and CSF P-tau181 with MMSE scores (ΔR^2^ = 0.024, *p* = 0.001) (Figures 5A,B), and sUA levels had no significant moderating effect on the association of CSF T-tau with MMSE scores (ΔR^2^ = 0.002, *p* = 0.381). The hyperphosphorylation of tau is the main cascade under Aβ42, so we explored whether sUA played a role in this key pathologic process of AD (Hardy and Higgins, 1992; Hardy and Selkoe, 2002). The analyses showed that high levels of sUA alleviated the association of CSF Aβ42 with CSF P-tau181 (ΔR^2^ = 0.037, *p* = 0.001) and CSF T-tau (ΔR^2^ = 0.044, *p* < 0.001) (Figure 5C, D).

## Discussion

UA is a result of purine metabolism, and it could be neuroprotective as a powerful antioxidant (mainly in blood) or neurotoxic as a prooxidant (mainly intracellular) or an inflammatory factor (Ames et al., 1981; Becker et al., 1991; So and Thorens, 2010; Kimura et al., 2021). As the results of our study, we tend to look at UA as a neuroprotective factor. Oxidative stress plays an important role in the pathological process of dementia, and UA has probable neuroprotective effects as a powerful antioxidant (Ames et al., 1981; Proctor, 2008). At the cellular or animal level, UA was found to protect hippocampal neurons from oxidative stress, and it can block nitrotyrosine formation in neurons and endothelial cells and maintain the integrity of the BBB (Hooper et al., 1998; Yu et al., 1998). Clinical studies have suggested that lower levels of UA could be a risk factor in multiple nervous system diseases, including AD (Schlesinger and Schlesinger, 2008; Peng et al., 2012; Abraham and Drory, 2014; Moccia et al., 2015; Scheepers et al., 2019; Guan et al., 2021). In AD, pathological biomarkers in the ATNX framework are associated with oxidative stress (Kaplow et al., 2020). UA could reduce the Aβ-induced tyrosine nitration of synaptophysin and the nitration and hyperphosphorylation of tau as a peroxynitrite scavenger (Tran et al., 2003; Zhang et al., 2006), and high levels of sUA could alleviate the effect of CSF Aβ42 and tau on cognitive decline (Ye et al., 2016).

UA could present different levels in different stages of CI. Taking AD as an example, first, several cross-sectional studies have observed higher levels of sUA in CI individuals than in CU individuals in preclinical AD or prodromal AD (Zuliani et al., 2018; Li et al., 2021). Second, longitudinal studies with sUA levels to predict the incidence of AD should pay attention to the stage of AD at baseline (Latourte et al., 2018). These results may not mean that sUA was a risk factor for AD, but there could be a compensatory increase in sUA levels for antioxidative effects confronting the early pathology in AD. In that light, high levels of sUA in the early phase of AD could be a biomarker of a high risk of dementia, which could suggest an attempted protective response. In addition, sUA could play different roles in different types of CI. Hyperuricaemia and gout could be linked to multiple comorbidities, including stroke, hypertension and diabetes, and these comorbidities are risk factors for dementia (Richette et al., 2020; Choi et al., 2022). The effect of UA on cognitive function primarily depends on the type of CI, and high sUA levels could lead to vascular dementia (VaD) but not AD (Cervellati et al., 2014; Latourte et al., 2018).

In our study, age, sex, *APOE* gene and GFR were independent influencing factors of sUA. These relationships suggested relatively low metabolism or insufficient nutrition in aged and female individuals. *APOE* is a metabolism-related gene, and the status of carrying *APOE* ε4 could be an interacting factor of UA. sUA excreted almost in the kidney, and renal dysfunction with a low GFR could lead to high levels of sUA.

There is a full spectrum of CI from CU to dementia, and we could take a panoramic view of the change in UA during the process of CI. We found a positive correlation between sUA levels and MMSE scores in AD patients, and the levels of sUA were lower in AD patients than in CU and non-AD patients, especially in the phase of dementia. The proportion of dementia and MCI was higher in group with lower levels of sUA. Additional analyses of UA and ATN biomarkers suggested the positive correlation of sUA with Aβ42, and sUA could alleviate the malignant effects of ATN biomarkers on cognitive function and Aβ42 on tau. The first increasing and then falling trend of sUA levels could be due to the early compensatory antioxidative function for Aβ42 and the later decompensation and self-exhaustion for anti-Aβ42 and the Aβ-tau pathological process or other amplifying vicious cascade in the later stage of AD. Therefore, sUA could be a protective factor of CI, and this effect predominantly acted on dementia stage and AD.

In the future, we should derive the exact causal relations between sUA and CI. Mendelian randomization (MR) studies could investigate the causal relationship of AD with UA, and more research based on familial AD cohorts is needed. There was a small quantity of data on hyperuricaemia, gout and hypouricaemia in our cohort. According to the present studies, high sUA levels were still protective not only in AD but also in VaD (Hong et al., 2015; Lu et al., 2016). Therefore, we should be committed to the management of UA in patients with AD and keep them in the status of relatively high levels of sUA based on the risk of cardiovascular diseases under control (Euser et al., 2009). According to the neuroprotective effects of UA, inosine, the precursor of urate, has been used in neurodegeneration disease as urate elevation therapy, and it could also be used in AD with hypouricaemia (Schwarzschild et al., 2021).

## Conclusion

UA is positively correlated with cognitive function and could be a protective factor of AD, especially in the advanced stage. Throughout the process of AD, sUA levels first increased and then decreased, and the peak was in the stage of MCI. The probable neuroprotective effects of sUA mainly act on Aβ42 and the downstream pathological cascade.

## Data availability statement

The original contributions presented in the study are included in this article/supplementary material, further inquiries can be directed to the corresponding authors.

## Ethics statement

The studies involving human participants were reviewed and approved by Institutional Review Board of Daping Hospital. Written informed consent to participate in this study was provided by the participants or their legal guardian/next of kin. Written informed consent was obtained from the individual(s), and minor(s)’ legal guardian/next of kin, for the publication of any potentially identifiable images or data included in this article.

## Author contributions

JG, Y-JW, and SH contributed to the study design, data interpretation, and writing of the report. JW, TL, YL, D-YF, Y-FT, Y-YS, G-HZ, D-WC, Y-RW, L-YC, SH, Y-JW, and JG contributed to the data collection, data analysis, data interpretation, and revision of the manuscript. All authors had full access to study data for interpretation and drafting of the report.

## Conflict of interest

The authors declare that the research was conducted in the absence of any commercial or financial relationships that could be construed as a potential conflict of interest.

## Publisher’s note

All claims expressed in this article are solely those of the authors and do not necessarily represent those of their affiliated organizations, or those of the publisher, the editors and the reviewers. Any product that may be evaluated in this article, or claim that may be made by its manufacturer, is not guaranteed or endorsed by the publisher.
